# Does dopamine block the spawning of the acroporid coral *Acropora tenuis*?

**DOI:** 10.1038/srep02649

**Published:** 2013-09-12

**Authors:** N. Isomura, C. Yamauchi, Y. Takeuchi, A. Takemura

**Affiliations:** 1 Department of Bioresources Engineering, Okinawa National College of Technology, 905 Henoko, Nago-City, Okinawa 905-2192, Japan; 2 Department of Chemistry, Biology and Marine Science, Faculty of Science, University of the Ryukyus, Senbaru 1, Nishihara, Okinawa 903-0213, Japan

## Abstract

Most corals undergo spawning after a particular moon phase, but how moon-related spawning is endogenously regulated in corals remains unknown. The objective of the present study was to evaluate whether dopamine (DA) affects spawning in *Acropora tenuis*. When pieces of four *A. tenuis* colonies were reared under a natural photoperiod and water temperature, spawning was observed after the predicted moon phase. After exposure to water containing DA at 0.1 μM, pieces of the same colonies only released 5 to 10 bundles. Co-treatment with DA and pimozide (D1 and D2 receptors antagonist), but not domperidone (D2 receptor antagonist), induced mass release of bundles from the colonies. A cross-experiment revealed high fertilization rates between the control colonies (95%) and between the control and DA-treated colonies (90%), suggesting that gametes developed normally in coral tissue. Therefore, DA appears to have an inhibitory effect on the spawning of *A. tenuis*.

Synchrony of reproductive events has been reported for multiple phyla and can confer biological advantages such as an increased chance of mating, decreased predation risk, and protection against hybridization^1^. Mass spawning is a well-known synchronous event that occurs in many hermatypic corals in not only tropical^2^,^3^ and subtropical^4^,^5^ areas, but also temperate regions^6^. Because this synchrony in corals occurs during a fixed season of the year in many areas, periodic changes in several environmental factors likely act as reliable cues in regulating the synchronous development and release of gametes. For example, previous studies have reported that steady increases in solar insolation^7^ and water temperature^4^ facilitate gamete growth, subsequently scheduling the prospective month(s) for spawning. In most cases, the simultaneous release of gametes is related to the peak period of lunar and tidal stimuli^8^,^9^ as well as to the timing of sunset^10^. Because spawnings exhibit temporal differences across areas, the strongest cue in a specific area appears to be effectively utilized by locally adapted corals.

Environmental factors are perceived by the sensory systems of corals and then transduced as endocrine signals, which actively regulate the synchronization of gametogenesis and spawning. Several reports have shown that coral tissues contain sex steroids^11^,^12^,^13^ as well as a gonadotropin-releasing hormone (GnRH)-like compound^14^. Estrogens also appear to be involved in reproduction, as seasonal variation in these steroid hormones occur in accordance with reproduction^13^. To date, however, the key factors (and how they interplay) regulating various reproductive events of corals remain unknown.

Dopamine (DA) is a catecholamine that acts as a neurotransmitter and hormone in animals. In marine invertebrates, DA reportedly controls phototaxis in the larvae of a bryzoan *Bugula neritina*^15^, settlement and metamorphosis in the Pacific oyster *Crassostrea gigas*^16^, and metamorphosis in the common slipper shell *Crepidula fornicata*^17^. In addition, previous studies have documented a positive correlation between gonadal growth and DA concentration in the neural system of the great scallop *Pecten maximus*^18^,^19^. In contrast, DA inhibited the growth and maturation of oocytes in the sea urchin *Strongylocentrotus* *nudus*^20^. These findings imply that DA plays some roles in stimulating or suppressing the reproductive events of invertebrates, although its physiological function appears to differ among species. In vertebrates, including certain fish and tetrapods, however, DA has an inhibitory effect on the release of gonadotropins from the pituitary^21^,^22^ and on the release of GnRH from its neuron^23^,^24^. Together, these previous results lead to the hypothesis that DA is likely to co-express with sex steroids and a GnRH-like immunoreactive compound in coral tissue during the synchronous growth and release of gametes and that these chemicals regulate the reproductive events of corals either in coordination or antagonistically.

The objective of the present study was to examine whether treatment with DA would affect the final step of reproductive events in the coral *Acropora tenuis* (Dana, 1846). This species is common in the Indo-Pacific, including Okinawa, Japan, where *A. tenuis* usually spawns about 1 h after sunset^25^. Like all *Acropora* species, *A. tenuis* produces bundles which mean packaged eggs and sperm sacks in each hermaphrodite polyp. These bundles appear in the polyp mouths (“setting”) about 30 min to 1 h before bundle release (“spawning”). We exposed colonies of *A. tenuis* to DA alone or in mixture with DA antagonists and compared gamete release, setting, and the occurrence of spawning among treatments. DA antagonists used in the present study were domperidone (DOM) and pimozide (PIM), which antagonize D2 receptor^26^ and D1 and D2 receptors^27^, respectively. We also performed cross-experiments among treatment groups, as the methodology for cross-experiments in the genus *Acropora* has been previously established^10^,^25^.

## Results

### Spawning

*Acropora tenuis* spawning results from several treatments in 2011 and 2012 are summarized in Table 1. Spawning of *A. tenuis* occurred under experimental conditions on 16 and 17 June 2011 (1 day before the full moon and on the day of the full moon). About 30–50 min after setting, all four control colony pieces had terminated their spawning within 30–40 min. With the exception of one colony (No. 1), the colonies (Nos. 2–4) of the DA-treatment group released only 5–10 bundles. After treatment with DA + DOM, two colonies (Nos. 1 and 3) failed to release any bundles, while the other two (Nos. 2 and 4) released very few (7–9) bundles.

**Figure 1:**
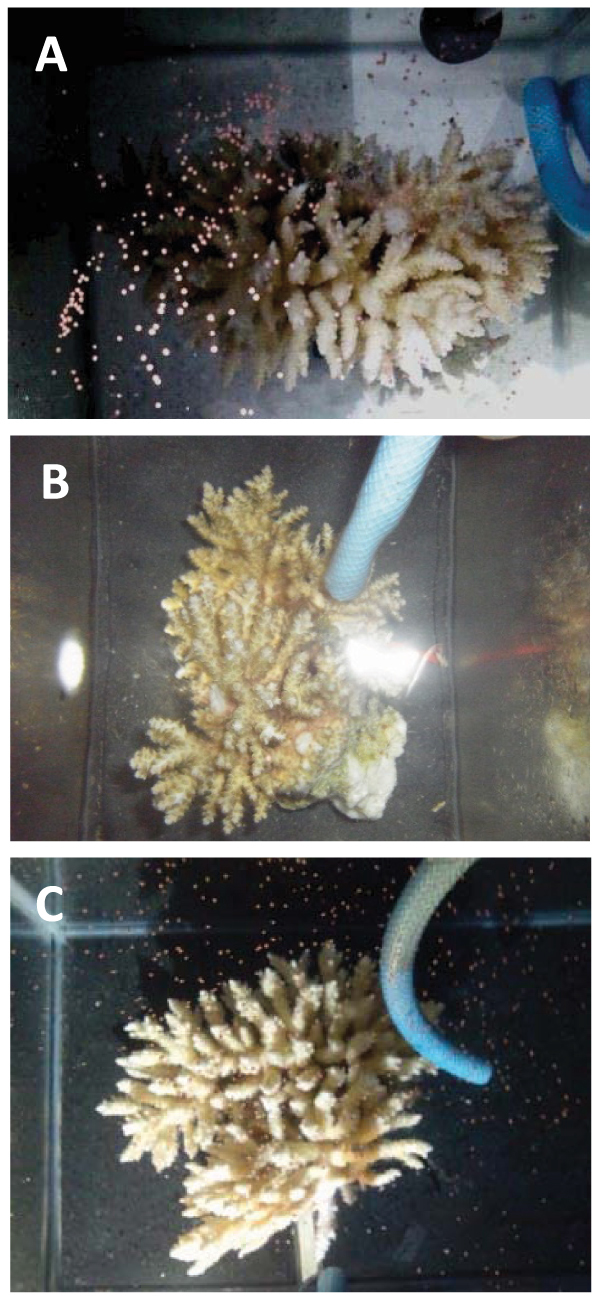
Photographs of spawning pieces from No. 3 colony of *Acropora tenuis* in 2012 under each treatment. (A), control; (B), DA (dopamine) treatment; (C), DA + PIM (dopamine antagonist, pimozide) treatment.

In 2012, spawning of *A. tenuis* was observed on 31 May (4 days before full moon) and 15 June (4 days after the last quarter moon). On 31 May, pieces of colony No. 1, either without (control) or with DA + PIM treatment, experienced spawning, whereas those treated with DA-alone released only 10 bundles. On 15 June, pieces of colony No. 3 in the control and DA + PIM treatments showed spawning, whereas pieces of the DA colony did not release bundles (Fig. 1). Pieces of colonies Nos. 2 and 4 did not spawn until 30 June, although the presence of developed gonads was confirmed.

### Fertilization ability and development

**Figure 2:**
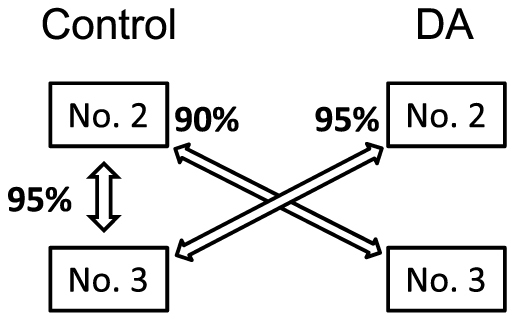
Relationship between crossed pair and fertilization rate from cross experiments in 2011. Crossed pair is connected by both directional arrows and values of percentage indicated by side of arrow head indicate fertilization rates. No. 2 and No. 3 show the number of colonies and DA shows dopamine treatment.

Figure 2 presents the fertilization rates from cross-experiments in 2011. A high fertilization rate (95%) was observed in the cross between controls (No. 2 × No. 3). The fertilization rates in crosses between control and DA-treatment colonies were similar to that of the control cross: 95% in the control (No. 3) × DA (No. 2) and 90% in the control (No. 2) × DA (No. 3). All embryos of each cross developed normally, as shown previously^28^, and became planula larvae within 72 h after insemination.

## Discussion

This is the first report to provide novel insight into regulatory mechanisms from major mass spawner *Acropora*, which is one of the widespread genera and shows multi-specific synchronous spawning around particular moon phase^2^,^3^. When pieces of matured *A. tenuis* colonies were continuously exposed to seawater containing DA during the final phase of gametogenesis, gamete release was not observed around the predicted spawning lunar phase. In contrast, pieces of control corals under the same rearing conditions experienced spawning around the predicted spawning lunar phase. These results indicate that DA has an inhibitory effect on gamete release from polyps. The role of DA in reproductive activity has been examined in various invertebrates^18^,^20^,^29^,^30^. High-performance liquid chromatography (HPLC) analyses have revealed that DA levels of the visceral ganglion are correlated with gonadal growth of the snail, *P. maximus*^18^. In contrast, DA treatment lowered the incorporation of labeled RNA and protein precursors into cultured gonads in the sea urchin *S. nudus*, suggesting an inhibitory effect of this neurotransmitter on oocyte growth and maturation^20^. These findings indicate that DA affects the reproductive activity of invertebrates but that its specific effect varies among species and/or growth stages. In the present study, matured gametes were confirmed in the polyps of DA-treated and control corals until the time of spawning. In addition, DA-treated gametes were successfully fertilized with control gametes, and the subsequent fertilization rates were high. This cross resulted in normal development of planula larvae within 72 h after insemination. Therefore, the inhibitory effect of DA likely occurs at the final stage of gamete release but not at the continuous stages of gametogenesis. Previous studies on seasonal variation in endogenous DA in the gonads of the oyster, *C. gigas*, have demonstrated that DA levels decreased during the active spawning season^31^ and increased thereafter^32^. When artificial spawning was induced in the matured gonad of *C. gigas* using UV-irradiated seawater, DA decreased during spawning^31^.

Co-treatment of colony pieces with DA and DOM (a D2 receptor antagonist^26^) failed to reduce the inhibitory effect of DA on the spawning of *A. tenuis*. In contrast, treatment with PIM, which antagonizes both the D1 and D2 receptors^27^, was able to diminish the inhibitory effect of DA. These results imply that the two DA antagonists vary in their effectiveness at blocking the effect of DA on the spawning of corals. Because no other experiments on invertebrates have examined the effects of DOM and/or PIM, whether antagonists exhibit an equivalent effect on other invertebrate species remains unclear. One explanation is that concentration of the antagonists influences on the present results, although we continuously exposed *A. tenuis* to the same concentration of DOM and PIM (0.1 nM). Period exposing to the antagonists may be also not ruled out, because DOM and PIM were exposed for one month and 10 days, respectively. Alternatively, the fact that only PIM could reduce the inhibitory effect of DA leads us to hypothesize that such function of DA is mediated through D1 receptor in *A. tenuis*. In this regard, we have cloned D1-like dopamine receptor cDNAs, but not D2 receptor, from the polyps of *A. tenuis* (see Supplementary Fig. S1–S6 online), supporting our hypothesis.

The presence of free and glucuronided estradiol (E2) has been documented in the tissue of the scleractinian coral *Euphyllia ancora* which is a gonochoric coral and it is not main member of the synchronous multispecific spawning event throughout the year, and the peaks in these steroids occur just prior to spawning^13^. The concentration of free E2 was higher than that of glucuronided E2 in the coral tissue during the spawning season, whereas a higher concentration of glucuronided E2 was detected in the seawater during coral spawning^13^. These authors suggested that increases in free E2 and its glucuronide in coral tissues are involved in gamete release and that E2 glucuronide released in seawater acts as a pheromone for chemical communication among coral colonies during spawning^13^. In accordance with fluctuation of these steroid hormones, the activity of aromatase, a key enzyme in converting testosterone to E2 that functions in oocyte growth in oviparous vertebrates^33^, was detected from January to June and dramatically increased toward the day of spawning in May^14^. Monthly measurement of steroid hormones in the tissue of *Montipora verrucosa* spawned in June and July using radioimmunoassay revealed that estrone (E1) and E2 increase in April and in February and March, respectively, indicating that estrogens are related to early stages of gametogenesis and protein synthesis^12^. In addition, it was reported that peaks in E2 occurred during the week prior to spawning and in E1 during spawning^12^. Similarly, estrogens are also likely involved in regulating gametogenesis and spawning in corals^12^,^14^. Here, we demonstrated that DA acts as an inhibitor of the final step of spawning in *A. tenuis.* Notably, DA levels in the gonads of the scallop *Patinopecten yessoensis* reportedly decreased after E2 administration^32^. Taken together, E2 may regulate DA levels in the gonads in not only this scallop, but also in corals.

Presumably, interplay of endogenous players (i.e., estrogens and DA) occurs at the final step of gametogenesis and spawning in corals. Synergistic action by endogenous players may accelerate the spawning of corals. However, E2 levels changed very little in relation to spawning in *M. verrucosa*^12^ and in the soft coral *Sinularia polydactyla*^11^. In addition, because GnRH-like immunoreactivity was detected from polyps of *E. ancora*^14^, corals may have dual neuroendocrine control of reproduction by GnRH and DA, which is the case for several adult teleosts^34^. Thus, a variety of regulatory mechanisms may control spawning across coral species. Further studies are necessary to better understand the physiological mechanisms of reproductive events in corals.

## Methods

### Coral maintenance and exposure experiments

Colonies of *A. tenuis* (20–30 cm in diameter) kept in tanks at Okinawa Churaumi Aquarium (26.69°N, 127.87°E) were used in the present study. After confirming gonadal development in these corals, colonies were transported to Sesoko Station, Tropical Biosphere Research Center, University of the Ryukyus, Japan (26.63°N, 127.86°E) on 20 May 2011 and 24 May 2012.

Experiments began 30 days and 10 days prior to the predicted spawning lunar phase in 2011 and 2012, respectively. The difference in exposure periods between two years was partially due to difficulty of predicted spawning date according to intercalary month and water temperature. DA and its antagonists, DOM (D2 receptor antagonist^26^) and PIM (D1 and D2 receptors antagonist^27^), were purchased from Sigma-Aldrich (St. Louis, MO, USA) and dissolved in distilled water as needed. Four colonies of *A. tenuis* (Nos. 1–4) were selected from the tanks of Okinawa Churaumi Aquarium at random in 2011 and 2012. Each colony was divided into four small pieces and reared separately in 20-L aquaria with running seawater and aeration under a natural photoperiod and water temperature at Sesoko Station. The colony pieces were continuously treated with DA (0.1 μM) or a mixture of DA (0.1 μM) and a DA antagonist. Vehicle-only treatments served as the control group.

Concentrations of DA and antagonists in each aquarium were constantly maintained by a Peristaltic Pump (AC-2110-2, ATTO, Tokyo, Japan). DOM at 0.1 nM and PIM at 0.1 nM were used as the DA antagonist in 2011 and 2012, respectively. Setting and spawning in all colony pieces were checked daily. Because the timings of setting and spawning of *A. tenuis* in Okinawa have previously been documented^25^, we also determined whether these timings occurred physiologically in the experimental groups. If spawning was not observed in the colonies, we checked the conditions of gametes in the colonies at 1 week after the main spawning of *A. tenuis*.

Treatments effects on fertilization ability were checked in the present study in 2011. Cross experiments were performed using gametes from two control pieces (Nos. 2 and 3) and two DA-treatment pieces (Nos. 2 and 3). Gamete bundles from each piece were collected in small tubes (8 mL, 60.582, ASSIST, Tokyo, Japan), and the gametes from two different pieces were mixed without separation of eggs and sperm in cross combinations. Three crosses (control [No. 2] × control [No. 3], control [No. 2] × DA [No. 3], and control [No.3] × DA [No.2]) were established, as the number of bundles produced from the DA-treatment pieces was very small. Sperm concentration was adjusted to more than 10^6^ sperms/mL. Self-fertilizing colonies did not occur in the present study. Twenty eggs from the cross experiments were separately transferred from each combination into Petri dishes 1 h after insemination. The numbers of fertilized and unfertilized eggs were scored at the 16-cell/morula stage 4–5 h after gamete mixing.

## Author Contributions

N.I. and A.T. designed the experiments. N.I. carried out the experiments and analyzed the data mainly. C.Y. and Y.T. contributed to the exposure experiments in Sesoko Station. Y.T. performed the molecular and genetic experiments. N.I. and A.T. wrote the manuscript that was edited by all other co-authors.

## Supplementary Material

Supplementary Information Supplementary Information

## Figures and Tables

**Table 1:** Date and time of setting and spawning and number of bundles released from pieces of *Acropora tenuis* under each treatment

					Treatment			
Year	Colony No.	Date of spawning	Start time of setting	Start time of spawning	control	dorpamine	dorpamine + DOM (domperidone)	dorpamine + PIM (pigmozide)
2011	1	17, Jun	19:15	20:00	Spawning	No bundles	No bundles	-
	2	16, Jun	19:15	20:00	Spawning	10	9	-
	3	16, Jun	19:15	20:10	Spawning	6	No bundles	-
	4	17, Jun	19:15	20:10	Spawning	5	7	-
2012	1	31, May	18:35	19:45	Spawning	10	-	Spawning
	2	(*No spawning in experimental duration*)			No bundles	No bundles	-	No bundles
	3	15, Jul	18:30	20:00	Spawning	No bundles	-	Spawning
	4	(*No spawning in experimental duration*)			No bundles	No bundles	-	No bundles

“Spawning”; more than 50 bundles, “-“; no treatment.

## References

[b1] Domeier M. L. & Colin P. L. Tropical reef fish spawning aggregations: Defined and reviewed. Bull. Mar. Sci. 60, 698–726 (1997).

[b2] Babcock R. C. *et al.* Synchronous spawnings of 105 scleractinian coral species on the Great Barrier Reef. Mar. Biol. 90, 379–394 (1986).

[b3] Harrison P. L. *et al.* Mass spawning in tropical reef corals. Science 223, 1187–1188 (1984). 10.1126/science.223.4641.1186 17742935

[b4] Hayashibara T. *et al.* Patterns of coral spawning at Akajima Island, Okinawa, Japan. Mar. Ecol. Prog. Ser. 101, 253–262 (1993).

[b5] Babcock R. C., Wills B. L. & Simpson C. J. Mass spawning of corals on a high latitude coral reef. Coral Reefs 13, 161–169 (1994).

[b6] Nozawa Y., Tokeshi M. & Nojima S. Reproduction and recruitment of scleractinian corals in a high-latitude coral community, Amakusa, southwestern Japan. Mar. Biol. 149, 1047–1058 (2006).

[b7] Van Woesik R., Lacharmoise F. & Koksal S. Annual cycles of solar insolation predict spawning times of Caribbean corals. Ecol. Lett. 9, 390–398 (2006). 16623724 10.1111/j.1461-0248.2006.00886.x

[b8] Krupp D. A. Sexual reproduction and early development of the solitary coral *Fungia scutaria* (Anthozoa: Scleractinia). Coral Reefs 2, 159–164 (1983).

[b9] Szmant A. F., Reutter M. & Riggs L. Sexual reproduction of *Favia fragum* (Esper): Lunar patterns of gametogenesis, embryogenesis and planulation in Puerto Rico. Bull. Mar. Sci. 37, 880–892 (1985).

[b10] Hatta M. *et al.* Reproductive and genetic evidence for a reticulate evolutionary history of mass-spawning corals. Mol. Biol. Evol. 16, 1607–1613 (1999). 10555292 10.1093/oxfordjournals.molbev.a026073

[b11] Slattery M., Hines G. A., Starmer J. & Paul V. J. Chemical signals in gametogenesis, spawning, and larval settlement and defense of the soft coral *Sinularia* *polydactyla*. Coral Reefs 18, 75–84 (1999).

[b12] Tarrant A. M., Atkinson S. & Atkinson M. J. Estrone and estradiol-17β concentration in tissue of the scleractinian coral, *Montipora verrucosa*. Comp. Biochem. Physiol. B. 122, 85–92 (1999). 10.1016/s1095-6433(98)10155-1 10216933

[b13] Twan W. H., Hwang J. S. & Chang C. F. Sex steroids in scleractinian coral, *Euphyllia* *ancora*: implication in mass spawning. Biol. Reprod. 68, 2255–2260 (2003). 12606339 10.1095/biolreprod.102.012450

[b14] Twan W. H. *et al.* The presence and ancestral role of GnRH in the reproduction of scleractinian coral, *Euphyllia ancora*. Endocrinology 147, 397–406 (2006). 16195400 10.1210/en.2005-0584

[b15] Pires A. & Woollacott R. M. Serotonin and dopamine have opposite effects on phototaxis in larvae of the Bryozoan *Bugula neritina*. Biol. Bull. 192, 399–409 (1997). 10.2307/1542749 28581842

[b16] Bonar D. B., Coon S. L., Walch M., Weiner R. M. & Fitt W. Control of oyster settlement and metamorphosis by endogenous and exogenous chemical cues. Bull. Mar. Sci. 46, 484–498 (1990).

[b17] Pechenik J. A., Wei L. & Cochrane D. E. Timing is everything: The effects of putative dopamine antagonists on metamorphosis vary with larval age and experimental duration in the prosobranch gastropod *Crepidula fornicate*. Biol. Bull. 202, 137–147 (2002). 11971809 10.2307/1543650

[b18] Paulet Y. M., Donval A. & Bekhadra F. Monoamines and reproduction in *Pecten maximus*, a preliminary approach. Invert. Rep. Dev. 23, 89–94 (1993).

[b19] Martinez G. & Revera A. Role of monoamines in the reproductive process of *Argopecten pupuratus*. Invert. Rep. Dev. 25, 167–174 (1993).

[b20] Khotimchenko Y. S. Effect of noradrenaline, dopamine and adrenolytics on growth and maturation of the sea urchin, *Strongylocentrotus* *nudus* Agassiz. Int. J. Invert. Rep. Dev. 4, 369–373 (1982).

[b21] Peter R. E. & Paulencu C. R. Involvement of the preoptic region in the gonadotropin release-inhibition in the goldfish. Neuroendocrinology 31, 133–141 (1980). 7393408 10.1159/000123064

[b22] Kah O., Dulka J. G., Dubourg P., Thibault J. & Peter R. E. Neuroanatomical substrate for the inhibition of gonadotrophin secretion in goldfish: existence of a dopaminergic preoptico-hypophyseal pathway. Neuroendocrinology 45, 451–458 (1987). 2886934 10.1159/000124774

[b23] Yu K. L. & Peter R. E. Dopaminergic regulation of brain gonadotropin-releasing hormone in male goldfish during spawning behaviour. Neuroendocrinology 52, 276–283 (1990). 2120609 10.1159/000125598

[b24] Yu K. L. & Peter R. E. Adrenergic and dopaminergic regulation of gonadotropin- releasing hormone release from goldfish preoptic-anterior hypothalamus and pituitary *in vitro*. Gen. Comp. Endocrinol. 85, 138–146 (1992). 1348716 10.1016/0016-6480(92)90181-i

[b25] Fukami H., Omori M., Shimoike K., Hayashibara T. & Hatta M. Ecological and genetic aspects of reproductive isolation by different spawning times in *Acropora* corals. Mar. Biol. 142, 679–684 (2003).

[b26] Dufour S. *et al.* Dopaminergic inhibition of reproduction in teleost fishes: ecophysiological and evolutionary implications. Ann. NY Acad. Sci. 1040, 9–22 (2005). 15891002 10.1196/annals.1327.002

[b27] Beninger R. J. *et al.* Effects of extinction, pimozide, SCH 23390, and metoclopramide on food-rewarded operant responding of rats. Psychopharmacology. 92, 343–349 (1987). 2957718 10.1007/BF00210842

[b28] Okubo N. & Motokawa T. Embryogenesis in the reef-building *Acropora* spp. Zool. Sci. 24, 1169–1177 (2007). 18271633 10.2108/zsj.24.1169

[b29] Fingerman M. Roles of neurotransmitters in regulating reproductive hormone release and gonadal maturation in decapod crustaceans. Invert. Rep. Dev. 31, 47–54 (1997).

[b30] Alfaro J., Zuniga G. & Komen J. Induction of ovarian maturation and spawning by combined treatment of serotonin and a dopamine antagonist, spiperone in *Litopenaeus stylirostris* and *Litopenaeus vannamei*. Aquaculture 236, 511–522 (2004).

[b31] Osada M., Matsutani K. & Nomura T. Implication of catecholamines during spawning in marine bivalve molluscs. Int. J. Invert. Rep. Dev. 12, 241–252 (1987).

[b32] Osada M. & Nomura T. Estrogen effect on the seasonal levels of catecholamines in the scallop *Patinopecten yessoensis*. Comp. Biochem. Physiol. C. 93, 349–353 (1989).

[b33] Mommsen T. P. Fish physiology. Volume XI. The physiology of developing fish. Part A. Eggs and larvae (eds Hoar, W. S. & Randall, D. J.) 348 (Academic Press, 1988).

[b34] Dufour S., Sebert M. E., Weltzien F. A., Rousseau K. & Pasqualini C. Neuroendocrine control by dopamine of teleost reproduction. J. Fish Biol. 76, 129–160 (2010). 20738703 10.1111/j.1095-8649.2009.02499.x

