# Supplementary material for: Does dopamine block the spawning of the acroporid coral Acropora tenuis?
Source: Sci Rep. 2013 Sep 12;3:2649. doi: 10.1038/srep02649 (PMC3770960; doi:10.1038/srep02649)
Supplement: Supplementary Information [file srep02649-s1.pdf]

Does dopamine block the spawning of the acroporid coral *Acropora tenuis*?

Naoko Isomura[1*], Chihiro Yamauchi[2], Yuki Takeuchi[2] & Akihiro Takemura[2]

[1]Department of Bioresources Engineering, Okinawa National College of Technology, 905 Henoko, Nago-City, Okinawa 905-2192, Japan

[2]Department of Chemistry, Biology and Marine Science, Faculty of Science, University of the Ryukyus, Senbaru 1, Nishihara, Okinawa 903-0213, Japan.

*Corresponding author: Naoko Isomura

Tel; +81-980-55-4135, Fax; +81-980-52-4252

E-mail; iso@okinawa-ct.ac.jp

```
  1 cccaacaaccaatcaaatccagtggctactgatccagacattggagacatcttggcagcc
    P  N  N  Q  S  N  P  V  A  T  D  P  D  I  G  D  I  L  A  A
 61 cttttagtcgccttgcttataatttgcacattaatcactaattgtttagtttgcgtaaca
    L  L  V  A  L  L  I  I  C  T  L  I  T  N  C  L  V  C  V  T
121 ttttacctatttaaggacctaaggacagtttgtcattatttgtcatcagtttgagcgcg
    F  Y  L  F  K  D  L  R  T  V  C  H  Y  F  V  I  S  L  S  A
181 gccgacattcttgttgctgtagtagctatgccggtttggtgtgcgctgcaactaacttcc
    A  D  I  L  V  A  V  V  A  M  P  V  W  C  A  L  Q  L  T  S
241 atgcattggtcgtttagtgactcactgaggacattttggaactgcatggacatactctgt
    M  H  W  S  F  S  D  S  L  R  T  F  W  N  C  M  D  I  L  C
301 ggaacagcttcaatcatgaatctcacagcagtgtcaattgatcgccatgctgccattact
    G  T  A  S  I  M  N  L  T  A  V  S  I  D  R  H  A  A  I  T
361 aagcctttcaactacccgtctgtcatgacttcttttcgcgctatttccatgataatcttt
    K  P  F  N  Y  P  S  V  M  T  S  F  R  A  I  S  M  I  I  F
421 gtgtggttttattccatagtggtttcgggattacgcttagcaacctggcacaccaaaaca
    V  W  F  Y  S  I  V  V  S  G  L  R  L  A  T  W  H  T  K  T
481 agttatatgcattttgtttctgcaaccagctttttattcctctcttcattatgataatc
    S  Y  M  H  F  V  S  A  T  S  F  F  I  P  L  F  I  M  I  I
541 atgtacacaagaatttatttggttgctaggaaacaagctcaccgaatgcgtaatggccgt
    M  Y  T  R  I  Y  L  V  A  R  K  Q  A  H  R  M  R  N  G  R
601 aactacgcaagtgatgtcaaagccgctaaaacgatcgctatattaatcggcttgttcgta
    N  Y  A  S  D  V  K  A  A  K  T  I  A  I  L  I  G  L  F  V
661 ttttgctggggtccatttttcgccattgtcctatctattgcgcatgatcagacggttgta
    F  C  W  G  P  F  F  A  I  V  L  S  I  A  H  D  Q  T  V  V
721 gttccttactcgctgttcaatgtcatcaaatggatggtgtactgcagttcttgtctgaac
    V  P  Y  S  L  F  N  V  I  K  W  M  V  Y  C  S  S  C  L  N
781 cccataatctactcttgtcttaatcgcaactacagacgtgcattgcgtaaactttgccaa
    P  I  I  Y  S  C  L  N  R  N  Y  R  R  A  L  R  K  L  C  Q
841 cgtgtcctcaaaaagcgagaaatggtcactgagacttcgtcg 882
    R  V  L  K  K  R  E  M  V  T  E  T  S  S
```

Figure S1. Nucleotide and deduced amino acid sequences of a partial ORF fragment of *Acropora tenuis* dopamine receptor-like cDNA (*At06753*). Oligonucleotide primers used to amplify the cDNA fragment by PCR were designed based on *Acropora digitifera* transcript sequence of aug_v2a.06753.t1. The cDNA was composed of 882 bases encoding a protein of 294 amino acids. The nucleotide numbers are shown to the left of each line. The putative transmembrane domains analyzed by a Kyte and Doolittle hydropathy plot are underlined with solid lines.

```
  1 gtggtgatgattctgagcgctgtgacaactgtgactggagaatggatatttggagcatgg
    V  V  M  I  L  S  A  V  T  T  V  T  G  E  W  I  F  G  A  W
 61 tgctgtcaggctgtagctgcagcgggtctgactttgtctttcatatcaattcttcacatg
    C  C  Q  A  V  A  A  A  G  L  T  L  S  F  I  S  I  L  H  M
121 agctgcctcagtatcgatcgatatattgcaattcagaaaccctttagctatgaatccatc
    S  C  L  S  I  D  R  Y  I  A  I  Q  K  P  F  S  Y  E  S  I
181 gtcactagaagaagagtggtgacggttttaatcctgatctgggtgtctggagttgttgtg
    V  T  R  R  R  V  V  T  V  L  I  L  I  W  V  S  G  V  V  V
241 cctaatattccgttagcagattttgaattcagggccgtgacatacggatgttcaagtgtg
    P  N  I  P  L  A  D  F  E  F  R  A  V  T  Y  G  C  S  S  V
301 gagtttgaaaacacccggcggtctttttctccttatattattttccttgtcggaatattt
    E  F  E  N  T  R  R  S  F  S  P  Y  I  I  F  L  V  G  I  F
361 gttgtcatacctttcgctgttatttgcttttcgaacgcagtggttttcaagacagctttt
    V  V  I  P  F  A  V  I  C  F  S  N  A  V  V  F  K  T  A  F
421 aatcacgcaagacagctgagcagagttgagaagagtttagagcggtcttcagcagatatt
    N  H  A  R  Q  L  S  R  V  E  K  S  L  E  R  S  S  A  D  I
481 tgcgaaagggaggagcaacagcaagcgaaaccacgcgtagaaaaccactcgttgaaaaga
    C  E  R  E  E  Q  Q  Q  A  K  P  R  V  E  N  H  S  L  K  R
541 gagatcaaatcagcgcacacctttgcgcttgtagtcggattattttgctatgttacatt
    E  I  K  S  A  H  T  F  A  L  V  V  G  L  F  L  L  C  Y  I
601 ccattttactcagtcggcacctacaggaagctagctcgatccgctaacgtacattctagt
    P  F  Y  S  V  G  T  Y  R  K  L  A  R  S  A  N  V  H  S  S
661 gatgtgcgcatcaccatgtgggttgctttcgccaattcttttatcaacccgattgtttat
    D  V  R  I  T  M  W  V  A  F  A  N  S  F  I  N  P  I  V  Y
721 ggcttgaggtattcaccgtttcgcaaagcttttaaattattatgtctagcccgctgtagt
    G  L  R  Y  S  P  F  R  K  A  F  K  L  L  C  L  A  R  C  S
781 gagcgacgccattctcacaactgcaagagaatcagttcttggagacgtgaaagggacggt
    E  R  R  H  S  H  N  C  K  R  I  S  S  W  R  R  E  R  D  G
841 accacgttatcaagtacttccgctctctag 870
    T  T  L  S  S  T  S  A  L  *
```

Figure S2. Nucleotide and deduced amino acid sequences of a partial ORF fragment of *Acropora tenuis* dopamine receptor-like cDNA (*At07635*). Oligonucleotide primers used to amplify the cDNA fragment by PCR were designed based on *Acropora digitifera* transcript sequence of aug_v2a.07635.t1. The cDNA was composed of 870 bases encoding a protein of 289 amino acids. The translation stopcodon is indicated by the asterisk (*). The nucleotide numbers are shown to the left of each line. The putative transmembrane domains analyzed by a Kyte and Doolittle hydropathy plot are underlined with solid lines.

```
  1 tatttatcgctcttggtcgtaattgctttgggaggtaatggtttgcttataacggttata
    Y  L  S  L  L  V  V  I  A  L  G  G  N  G  L  L  I  T  V  I
 61 ctcaagagaaaactccttcaaaaagttcattacttcttcatacttagtctggcggcgtct
    L  K  R  K  L  L  Q  K  V  H  Y  F  F  I  L  S  L  A  A  S
121 aatttcttaaacgccttttttaagatacctaccacgattttaggacgctttgatcgaaac
    N  F  L  N  A  F  F  K  I  P  T  T  I  L  G  R  F  D  R  N
181 tggtaccctaatcacacaatatgctacttcaccacccccctgggtgtgctgtttggcgcg
    W  Y  P  N  H  T  I  C  Y  F  T  T  P  L  G  V  L  F  G  A
241 gcatctgtgttcagcctatcagcggtagctatcaacagataccttgtgatttcctcgccg
    A  S  V  F  S  L  S  A  V  A  I  N  R  Y  L  V  I  S  S  P
301 cttaactattctgatcgaatgccaccgatgttggccaaaagcattttggctggaatttgg
    L  N  Y  S  D  R  M  P  P  M  L  A  K  S  I  L  A  G  I  W
361 ttcgcaagtttttctttggccgtccctcctgtcatgtggagagagaaggaagccatttgc
    F  A  S  F  S  L  A  V  P  P  V  M  W  R  E  K  E  A  I  C
421 cgaagcggtagaatttccaaagaacattacacctcggaaatgctttactttttcttggcc
    R  S  G  R  I  S  K  E  H  Y  T  S  E  M  L  Y  F  F  L  A
481 ttgtggctgtttgtcatcattgttccttccattgttatgagcatttcttacgtgaagatc
    L  W  L  F  V  I  I  V  P  S  I  V  M  S  I  S  Y  V  K  I
541 tttcttattgcgcgctaccacgcgttgcaaatagacacgcgaaatcaagcattcgcagat
    F  L  I  A  R  Y  H  A  L  Q  I  D  T  R  N  Q  A  F  A  D
601 tgccaacaaacaaagcgaaggaggaaagacttgaaagctgctgtagttctagcagtgatc
    C  Q  Q  T  K  R  R  R  K  D  L  K  A  A  V  V  L  A  V  I
661 ggaggaatcttcattatttgctggattcctttcttcgttgtacagactattcacaagttt
    G  G  I  F  I  I  C  W  I  P  F  F  V  V  Q  T  I  H  K  F
721 ggaaagggaaaaattagttctatttacttcaacatatttctctgcgtcatgtac 776
    G  K  G  K  I  S  S  I  Y  F  N  I  F  L  C  V  M  Y
```

Figure S3. Nucleotide and deduced amino acid sequences of a partial ORF fragment of *Acropora tenuis* dopamine receptor-like cDNA (*At08250*). Oligonucleotide primers used to amplify the cDNA fragment by PCR were designed based on *Acropora digitifera* transcript sequence of aug_v2a.08250.t1. The cDNA was composed of 776 bases encoding a protein of 258 amino acids. The nucleotide numbers are shown to the left of each line. The putative transmembrane domains analyzed by a Kyte and Doolittle hydropathy plot are underlined with solid lines.

```
1    atggccgtcagcaattctaccgcaatgcccccaacagatatagacggagtttgccaacaa
     M  A  V  S  N  S  T  A  M  P  P  T  D  I  D  G  V  C  Q  Q
61   gcaacgaagcccgagtttattgtttacagcgtttttttggtgctaatcatgttagccaca
     A  T  K  P  E  F  I  V  Y  S  V  F  L  V  L  I  M  L  A  T
121  ttgtttggaaatgtgttagttataaccgcggtttatcattttcatcgcttgcgacgaatg
     L  F  G  N  V  L  V  I  T  A  V  Y  H  F  H  R  L  R  R  M
181  actaatttcttcatcatctctctggccgtttccgatcttctcgttgcactaggtcatctg
     T  N  F  F  I  I  S  L  A  V  S  D  L  L  V  A  L  G  H  L
241  cctctccgtattgatcaaagcgttcacaacaataactggtgtttcgacaagacgcccaat
     P  L  R  I  D  Q  S  V  H  N  N  N  W  C  F  D  K  T  P  N
301  gatgtaacaacctgcgcgtattggatagcaatggacactgtgttcagctgtgcgtccatt
     D  V  T  T  C  A  Y  W  I  A  M  D  T  V  F  S  C  A  S  I
361  tgcaatctggttgtcatatcaattgatcgttttctagccatcaccaaacctttcgaatac
     C  N  L  V  V  I  S  I  D  R  F  L  A  I  T  K  P  F  E  Y
421  caaaacaggatgaccaagcgtgttggcttttcgctcatcgcttttgtttgggtctacgct
     Q  N  R  M  T  K  R  V  G  F  S  L  I  A  F  V  W  V  Y  A
481  ttactatggggtgtccttagcctggttgattggacaagggcagatcctaacgccactcac
     L  L  W  G  V  L  S  L  V  D  W  T  R  A  D  P  N  A  T  H
541  aggcatatcttcgtcatcgtaaaaaatcaaacaaatgagcgagcgtgcggcaagaaagac
     R  H  I  F  V  I  V  K  N  Q  T  N  E  R  A  C  G  K  K  D
601  aaggtgtattacactactgcaatggctgttgcgctatttttaccgcttctcatagtcatc
     K  V  Y  Y  T  T  A  M  A  V  A  L  F  L  P  L  L  I  V  I
661  gccacgtatgcctgcgttttccgcgtagcgtttacgcaggcgaaagcggtagccctactt
     A  T  Y  A  C  V  F  R  V  A  F  T  Q  A  K  A  V  A  L  L
721  gatccaaccaaaggaaagagacacattttgcgcgaattgaaagcgaccaaaactatcgct
     D  P  T  K  G  K  R  H  I  L  R  E  L  K  A  T  K  T  I  A
781  gttgttatcggggtattcatggtgtgttggctaccgagttttattatcattgtattgagt
     V  V  I  G  V  F  M  V  C  W  L  P  S  F  I  I  I  V  L  S
841  ctatggtgtcaggactgtttcaagccgtttttggacaacaaaaatctttctctctctata
     L  W  C  Q  D  C  F  K  P  F  L  D  N  K  N  L  S  L  S  I
901  aggatcatttttgtgaccattttgccggtcataaacagttctctaaatccggtgatttat
     R  I  I  F  V  T  I  L  P  V  I  N  S  S  L  N  P  V  I  Y
961  acagttttcaaccaagagttccgaatggccttcagtcgaatgctctgcagaggtcgaact
     T  V  F  N  Q  E  F  R  M  A  F  S  R  M  L  C  R  G  R  T
1021 ccacggagcgcagctgacgttgaattctcggttaccgaacaaacaaattctgcaacgcga
     P  R  S  A  A  D  V  E  F  S  V  T  E  Q  T  N  S  A  T  R
1081 gtgaaagtgcaaactacaggaaaaagcagaccaaaagcctggttcaatatgaacggtgga
     V  K  V  Q  T  T  G  K  S  R  P  K  A  W  F  N  M  N  G  G
1141 ggcgatgcgagcaggtag 1158
     G  D  A  S  R  *
```

Figure S4. Nucleotide and deduced amino acid sequences of a complete ORF fragment of *Acropora tenuis* dopamine receptor-like cDNA (*At14160*). Oligonucleotide primers used to amplify the cDNA fragment by PCR were designed based on *Acropora digitifera* transcript sequence of aug_v2a.14160.t1. The cDNA was composed of 1158 bases encoding a protein of 385 amino acids. The translation stopcodon is indicated by the asterisk (*). The nucleotide numbers are shown to the left of each line. The putative transmembrane domains analyzed by a Kyte and Doolittle hydropathy plot are underlined with solid lines.

```
1   gtcactgcgatctgtggaaacttactcgtatgcctggccatttcactgaacaggcgctta
    V  T  A  I  C  G  N  L  L  V  C  L  A  I  S  L  N  R  R  L
61  cgaaagaccaccaactacttcatattctccctggcgatttctgatcttttgacagcatca
    R  K  T  T  N  Y  F  I  F  S  L  A  I  S  D  L  L  T  A  S
121 tgctcaatgtcttttgatgttcaagttcttttgcaaccattgggctggaatcacggagag
    C  S  M  S  F  D  V  Q  V  L  L  Q  P  L  G  W  N  H  G  E
181 tttgtctgcaatttctggaccttcgtttatctgatcgctgcgccgacctccatacttaat
    F  V  C  N  F  W  T  F  V  Y  L  I  A  A  P  T  S  I  L  N
241 cttatggcggtcagcattgacaggtaccaagcaatcagtaatcctctccgatactacgac
    L  M  A  V  S  I  D  R  Y  Q  A  I  S  N  P  L  R  Y  Y  D
301 aaaatgagaccgagacgtgctatggctatcatagcagcaatttggctctactccttcgcg
    K  M  R  P  R  R  A  M  A  I  I  A  A  I  W  L  Y  S  F  A
361 tttacagttgctgggatggctggttggccctattatgaacaaagtgttcgggatggaatg
    F  T  V  A  G  M  A  G  W  P  Y  Y  E  Q  S  V  R  D  G  M
421 tgctattttaatatcagtccttactattctgtagtgagctcggcgatgaatttcattttc
    C  Y  F  N  I  S  P  Y  Y  S  V  V  S  S  A  M  N  F  I  F
481 cccacaatggtgatgtgtgtgatctatttcaaaatctatctgatcgctcgcgcgcatgca
    P  T  M  V  M  C  V  I  Y  F  K  I  Y  L  I  A  R  A  H  A
541 caacggcttgtgcagcatgaggtcccggtcaccaccgcagcaacatcttgctccaacgag
    Q  R  L  V  Q  H  E  V  P  V  T  T  A  A  T  S  C  S  N  E
601 gattctggcactataacctctgaaaagaaacgcctgaaaagaaatattaaggcggcaaag
    D  S  G  T  I  T  S  E  K  K  R  L  K  R  N  I  K  A  A  K
661 acaatcgccattattgtgtcaacttttctcctatgttgggtccctttcactttggtttca
    T  I  A  I  I  V  S  T  F  L  L  C  W  V  P  F  T  L  V  S
721 accattacttccctctgtcaatactgcatcgccaatttcgcggtagttattaactcacta
    T  I  T  S  L  C  Q  Y  C  I  A  N  F  A  V  V  I  N  S  L
781 cttgtggtggcgtacatgaactccgcactcaac 813
    L  V  V  A  Y  M  N  S  A  L  N
```

Figure S5. Nucleotide and deduced amino acid sequences of a partial ORF fragment of *Acropora tenuis* dopamine receptor-like cDNA (*At17552*). Oligonucleotide primers used to amplify the cDNA fragment by PCR were designed based on *Acropora digitifera* transcript sequence of aug_v2a.17552.t1. The cDNA was composed of 813 bases encoding a protein of 271 amino acids. The nucleotide numbers are shown to the left of each line. The putative transmembrane domains analyzed by a Kyte and Doolittle hydropathy plot are underlined with solid lines.

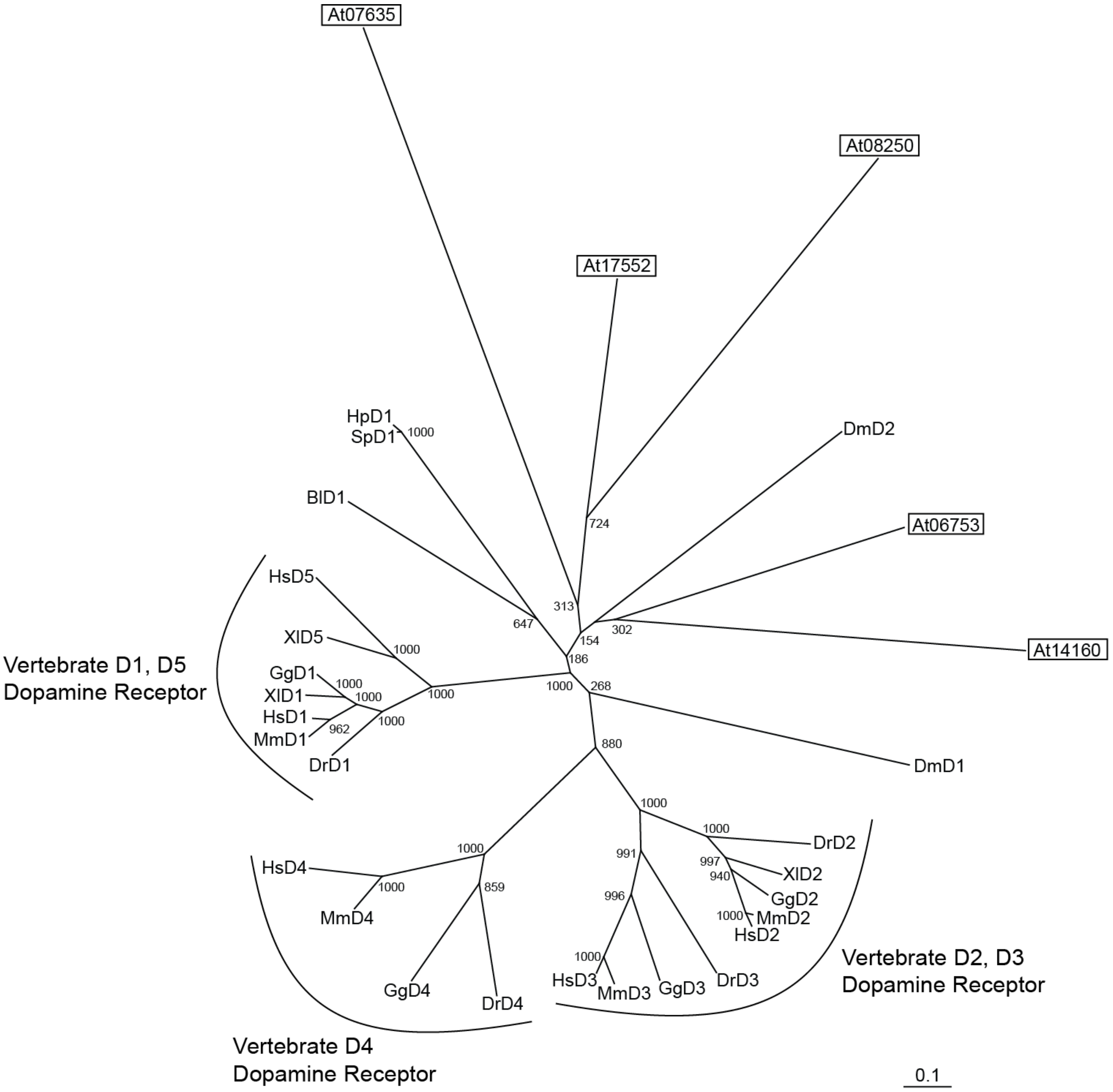

Figure S6. Unrooted phylogenetic tree of dopamine receptor proteins. The neighbor-Joining method and CLUSTAL W (http://clustalw.ddbj.nig.ac.jp/top-e.html) were used to construct the phylogenetic tree. One thousand bootstrap repetitions were performed, and bootstrap values are shown at the nodes. The scale bar refers to a phylogenetic distance of 0.1 amino acid substitution per site. The position of the Acropora tenuis dopamine receptor-like proteins were indicated by box. The accession numbers for dopamine receptor proteins used in the phylogenetic trees analysis are as follows: BlD1 (*Branchiostoma lanceolatum* D1, CAA06536); DmD1 (*Drosophila melanogaster* D1, P41596); DmD2 (*Drosophila melanogaster* D2, Q24563); DrD1 (*Danio rerio* D1, NP_001129448); DrD2 (*Danio rerio* D2, NP_898891); DrD3 (*Danio rerio* D3, NP_898890); DrD4 (*Danio rerio* D4, NP_001012634); GgD1 (*Gallus gallus* D1, NP_001138320); GgD2 (*Gallus gallus* D2, NP_001106761); GgD3 (*Gallus gallus* D3, ACR48171); GgD4 (*Gallus gallus* D4, NP_001136321); HpD1 (*Hemicentrotus pulcherrimus* D1, BAJ14803); HsD1 (*Homo sapiens* D1, NP_000785); HsD2 (*Homo sapiens* D2, AAB26819); HsD3 (*Homo sapiens* D3, P35462); HsD4 (*Homo sapiens* D4, NP_000788); HsD5 (*Homo sapiens* D5, AAA52329); MmD1 (*Mus musculus* D1,

NP_034206); MmD2 (*Mus musculus* D2, NP_034207); MmD3 (*Mus musculus* D3, NP_031903); MmD4 (*Mus musculus* D4, NP_031904); SpD1 (*Strongylocentrotus purpuratus* D1, XP_003726379); XlD1 (*Xenopus laevis* D1, P42289); XlD2 (*Xenopus laevis* D2, CAA42088); XlD5 (*Xenopus laevis* D5, P42290).
